# Supplementary material for: Discovery and application of insertion-deletion (INDEL) polymorphisms for QTL mapping of early life-history traits in Atlantic salmon
Source: BMC Genomics. 2010 Mar 8;11:156. doi: 10.1186/1471-2164-11-156 (PMC2838853; doi:10.1186/1471-2164-11-156)
Supplement: Additional file 2 — Information on developed 76 locus single-run INDEL panel in Atlantic salmon. Information on fluorescence labeling, primer concentrations, PCR pooling and links to alignments, INDEL motifs and GENESCAN (Burge and Karlin 1997) predictions of genes/exons are available in html format. [file 1471-2164-11-156-S2.ZIP › Additionalfile2/snpsummary8570.html]

```
Cluster 2694 Contig 1

prev  Summary    Contig List  next
```

Size of Consensus sequence = 723

Number of sequences = 21

Minimum redundancy = 6

Key

A gi|24343561|gb|CA042641.1|CA042641 ssalplnb504043 gut Salmo salar cDNA, mRNA sequence  
B gi|29318621|gb|CB506975.1|CB506975 ssaltc022067\_rev reproductive Salmo salar cDNA, mRNA sequence  
C gi|29314667|gb|CB503441.1|CB503441 ssalplnb510262\_rev gut Salmo salar cDNA, mRNA sequence  
D gi|117827442|gb|EG900138.1|EG900138 EST\_ssal\_evf\_39026 ssalevf mixed\_tissue Salmo salar cDNA Salmo salar cDNA clone ssal\_evf\_552\_029\_fwd 3', mRNA sequence  
E gi|117853900|gb|EG926596.1|EG926596 EST\_ssal\_evf\_29094 ssalevf mixed\_tissue Salmo salar cDNA Salmo salar cDNA clone ssal\_evf\_538\_223\_fwd 3', mRNA sequence  
F gi|117827444|gb|EG900140.1|EG900140 EST\_ssal\_evf\_39027 ssalevf mixed\_tissue Salmo salar cDNA Salmo salar cDNA clone ssal\_evf\_552\_029\_rev 5', mRNA sequence  
G gi|117853899|gb|EG926595.1|EG926595 EST\_ssal\_evf\_29093 ssalevf mixed\_tissue Salmo salar cDNA Salmo salar cDNA clone ssal\_evf\_538\_223\_rev 5', mRNA sequence  
H gi|117860415|gb|EG933111.1|EG933111 EST\_ssal\_evf\_34957 ssalevf mixed\_tissue Salmo salar cDNA Salmo salar cDNA clone ssal\_evf\_546\_201\_fwd 3', mRNA sequence  
I gi|117860416|gb|EG933112.1|EG933112 EST\_ssal\_evf\_34958 ssalevf mixed\_tissue Salmo salar cDNA Salmo salar cDNA clone ssal\_evf\_546\_201\_rev 5', mRNA sequence  
J gi|117468372|gb|EG800591.1|EG800591 EST\_ssal\_evd\_57314 ssalevd thymus Salmo salar cDNA Salmo salar cDNA clone ssal\_evd\_577\_114\_rev 5', mRNA sequence  
K gi|89883604|gb|DY739727.1|DY739727 EST\_ssal\_rgb2\_95466 ssalrgb2 mixed\_tissue Salmo salar cDNA Salmo salar cDNA clone ssal\_rgb2\_656\_136\_fwd 3', mRNA sequence  
L gi|89883605|gb|DY739728.1|DY739728 EST\_ssal\_rgb2\_95467 ssalrgb2 mixed\_tissue Salmo salar cDNA Salmo salar cDNA clone ssal\_rgb2\_656\_136\_rev 5', mRNA sequence  
M gi|117482759|gb|EG814976.1|EG814976 EST\_ssal\_evd\_34799 ssalevd thymus Salmo salar cDNA Salmo salar cDNA clone ssal\_evd\_546\_142\_rev 5', mRNA sequence  
N gi|117825758|gb|EG898454.1|EG898454 EST\_ssal\_evf\_37509 ssalevf mixed\_tissue Salmo salar cDNA Salmo salar cDNA clone ssal\_evf\_549\_384\_rev 5', mRNA sequence  
O gi|117468373|gb|EG800592.1|EG800592 EST\_ssal\_evd\_57315 ssalevd thymus Salmo salar cDNA Salmo salar cDNA clone ssal\_evd\_577\_114\_fwd 3', mRNA sequence  
P gi|117568638|gb|EG889614.1|EG889614 EST\_ssal\_evf\_47554 ssalevf mixed\_tissue Salmo salar cDNA Salmo salar cDNA clone ssal\_evf\_563\_218\_rev 5', mRNA sequence  
Q gi|117568639|gb|EG889615.1|EG889615 EST\_ssal\_evf\_47555 ssalevf mixed\_tissue Salmo salar cDNA Salmo salar cDNA clone ssal\_evf\_563\_218\_fwd 3', mRNA sequence  
R gi|117482758|gb|EG814975.1|EG814975 EST\_ssal\_evd\_34798 ssalevd thymus Salmo salar cDNA Salmo salar cDNA clone ssal\_evd\_546\_142\_fwd 3', mRNA sequence  
S gi|24344761|gb|CA043802.1|CA043802 ssalplnb510262 gut Salmo salar cDNA, mRNA sequence  
T gi|117532781|gb|EG864226.1|EG864226 EST\_ssal\_eve\_57095 ssaleve thyroid Salmo salar cDNA Salmo salar cDNA clone ssal\_eve\_577\_309\_fwd 3', mRNA sequence  
U gi|117532782|gb|EG864227.1|EG864227 EST\_ssal\_eve\_57096 ssaleve thyroid Salmo salar cDNA Salmo salar cDNA clone ssal\_eve\_577\_309\_rev 5', mRNA sequence

4 SNPs detected

A B C D E F G H I J K L M N O P Q R S T U  cosegregation weighted

453 G G A G G G G G G A G G A A A A A A A G G   1/4 25.00
610 . . T - - - - - - T T T T T T T T T T - -   3/4 67.86
611 . . C - - - - - - C C C C C C C C C C - -   3/4 67.86
612 . . C - - - - - - C C C C C C C C C C - -   3/4 67.86
